# The Effect of Number of Teeth and Chewing Ability on Cognitive Function of Elderly in UAE: A Pilot Study

**DOI:** 10.1155/2017/5732748

**Published:** 2017-11-14

**Authors:** Zahra Seraj, Dana Al-Najjar, Mohammed Akl, Noorelrahman Aladle, Yousif Altijani, Ahmed Zaki, Sausan Al Kawas

**Affiliations:** College of Dental Medicine, University of Sharjah, Sharjah, UAE

## Abstract

Cognitive decline is one of the major causes of disability among the aging population. The aim of this study was to explore the relationship between oral health parameters (number of teeth, chewing ability, and presence of a denture) and cognitive function in the elderly across the UAE. Fifty persons (age ≥ 60; 71.26 ± 10.23) were enrolled in the study. Cognitive status was assessed using the standardized mini-mental state examination (SMMSE) and accordingly, cognitively normal subjects scoring ≥24 were considered as the control group and cognitively impaired individuals scoring ≤23 were considered as the low scoring group. Chewing ability was examined, number of teeth was noted, and demographical data was collected. The results of this pilot study showed that individuals with low SMMSE scores were significantly less educated (*P* < 0.01) and had fewer number of remaining teeth (*P* < 0.05) and impaired chewing ability (*P* < 0.05). These results demonstrate a significant link between the number of teeth, chewing ability, and cognitive function. However, this pilot study had its limitations and was the first of its kind in the UAE and Gulf region; therefore, future research addressing the limitations is needed to further explore this association.

## 1. Introduction

On a global scale, according to the United Nations, by mid-century, the number of people over 60 years will represent 32% of the world population [1]. Considering the UAE, the proportion of elderly persons aged 60 years and above in the year 2000 was 5.1% and is expected to increase to 23.6% by 2025 [2]. The UAE provides a unique population in which the UAE nationals make up 11.6% of the population while the other 88.4% are expatriates [3], many of whom have been born in the UAE or have lived there for generations. Thus, it provides a controlled environment in which we can study both UAE nationals and expatriates. This, in addition to the rise in life expectancy from 74 years to 78 years [2], means there is an increased need to address age-related chronic diseases within the UAE.

Dementia, a neurocognitive disorder, is a broad term used to describe a range of symptoms associated with a decline in cognitive function and is one of the most common age-associated diseases. It is characterized by memory loss, neurological symptoms, disorientation, impaired judgement, personality changes, and loss of motor function [4]. Diagnosing dementia remains a challenge for physicians due to the symptom overlap with many other conditions such as depression, vitamin deficiencies, and thyroid dysfunction [5]. There is no cure for dementia and treatment remains to address the patients' symptoms and attempts to improve their quality of life and that of their families. Therefore, an understanding of early decline in cognitive function and the associated predisposing risk factors is becoming more important, shifting the focus towards prevention and delayed onset of disease.

Among the literature, links have been established between certain systemic factors such as diabetes [6, 7] and cardiovascular diseases [8] and their bidirectional association with poor dental and periodontal health. The links are increasingly being investigated as a possible means to address risk factors. Accordingly, the potential relationship between oral health and cognitive function has become a topic of interest. A wide range of studies have been conducted to assess the link between oral health conditions such as tooth loss [5, 9–19], impaired chewing ability [6, 20–26], and the absence of a denture [27, 28] in relation to cognitive impairment. The rationale is based upon the sensory and motor cortical remapping hypothesis, relating tooth loss and impaired masticatory ability to neuroanatomical and chemical changes that occur in the brain due to the reduction in sensory input and cortical blood flow [25, 26]. Additionally, studies have linked dental status and the use of dentures to improved nutritional intake [27, 29–38] and have established a relationship between low dietary intake of omega-3 fatty acids [38, 40], antioxidants [41, 42], and vitamin B12 [40–42] and an increased risk for cognitive decline [43–45]. Systemic factors such as hypertension [46–48], obesity [49, 50], and hypercholesterolemia [39] were also found to be risk factors. Periodontal disease and periodontal inflammatory blood markers have also been investigated in relation to cognitive decline [51].

To the best of our knowledge, no similar studies have been conducted within the Middle East or Gulf region. Therefore, the objectives of this pilot study are to (1) examine the relationship between the number of remaining teeth and cognitive ability, (2) examine the relationship between chewing status and cognitive ability, (3) examine the relationship between the presence of a denture and cognitive ability, and (4) test whether other demographic characteristics may be related to cognitive function.

## 2. Materials and Method

### 2.1. Participants

The Medical Ethics Committee of the University of Sharjah, UAE, approved data collection for this study. The sample consisted of 50 participants, 25 males and 25 females, all 60 years and above within the UAE. Written informed consent was obtained from each of the participants prior to their involvement in the study. The exclusion criteria included any disorders interfering with psychometric assessment such as severe blindness or terminal illness and/or conditions such as depression or history of cerebrovascular accident.

### 2.2. Assessment of Cognitive Mental Status

The Standardized Mini-Mental State Examination test (SMMSE) [52] was translated into Arabic using a forward-backward approach and was used to measure participants' global cognitive status. The SMMSE (score range 0–30) is the most common instrument used as a screening tool for cognitive function. It tests orientation, registration, short-term memory, language use, comprehension, and basic motor skills. According to the examination guidelines, participants were considered part of the low scoring group at a score of 23 or below, while those scoring 24 and above represented normal cognitive function (SMMSE ≤ 23 or SMMSE ≥ 24, resp.).

### 2.3. Assessment of Oral Health Parameters

Three parameters were explored and data was collected for each participant, number of teeth present, presence/absence of denture, and chewing ability using* the Index of Chewing Ability (ICA) *[53] which was translated into Arabic using a forward-backward approach. The ICA consists of five yes/no questions (score range 0–5) based on the ability to chew certain foods. Accordingly, those scoring less than 5 were considered to have impaired chewing ability while a score of 5 indicated competent chewing ability (ICA ≤ 4 or ICA = 5, resp.).

### 2.4. Other Recorded Variables

Questionnaires were administered through interviews to collect the data. Demographic variables (age, gender, and education level), lifestyle variables (smoking status), and the presence of chronic medical diseases such as cancer, cerebrovascular disease, myocardial infarction, diabetes mellitus, and hypertension were noted.

### 2.5. Statistical Analysis

Statistical analyses were performed using SPSS® Ver. 24.0 for Mac OS X. Clinical variables were analysed using independent *t*-tests and the chi-square test. The Pearson correlation was conducted to analyse correlations between cognitive impairment, dental health status, chewing ability, and denture presence. Two-tailed *P* values were calculated in all the analyses. Differences were considered statistically significant at *P* < 0.05.

## 3. Results

The subjects were divided into two groups according to their SMMSE scores (low scoring, SMMSE ≤ 23, *n* = 31 or SMMSE ≥ 24, *n* = 19; control).  Table 1 shows the demographic characteristics of the subjects included in the study and the summary statistics of oral health variables and SMMSE scores are listed in Table 2. The subjects with low SMMSE scores were found to be significantly less educated, had fewer remaining teeth, and, according to the ICA, had impaired chewing ability. It was also noted among nationals. No other differences were observed in all the other characteristics.

**Figure 1:**
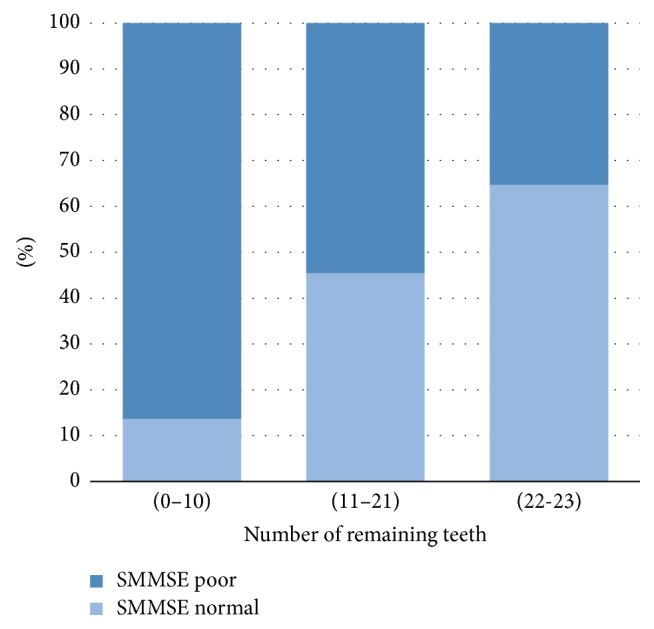
Prevalence of normal SMMSE or low SMMSE score according to the number of remaining teeth.

Figure 1 shows the prevalence of a normal or low SMMSE score and the number of teeth remaining. The number of remaining teeth (range: 0–32) was categorized into 3 (22–32, 11–21, and 0–10). The number of remaining teeth was significantly associated with low SMMSE scores (*r* = +0.465, *P* < 0.05). The prevalence of low SMMSE score was 86.4% in subjects with 0–10 remaining teeth, 54.5% in those with 11–21 teeth remaining, and 35.3% in those with 22–32 remaining teeth.

**Figure 2:**
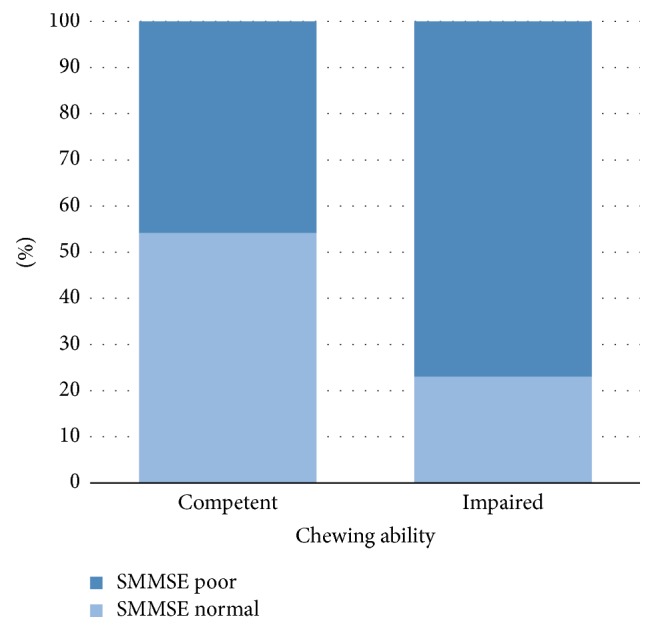
Prevalence of normal SMMSE or low SMMSE score according to chewing ability.

Figure 2 shows the prevalence of a normal or low SMMSE score and chewing ability. Individuals' chewing ability was classified as either competent or impaired. A significant association was observed between chewing ability and the prevalence of low SMMSE scores (*r* = 0.320, *P* < 0.05). The prevalence of low SMMSE score was 45.8% in persons with competent chewing ability and 76.9% in those with impaired chewing ability.

## 4. Discussion

This cross-sectional pilot study was designed to explore the relationship between number of remaining teeth, chewing ability, denture presence, and cognitive function in an elderly UAE population.

The study revealed that the prevalence of a low SMMSE score or poor cognition was significantly greater in association with fewer teeth remaining (*P* < 0.05) (Figure 1). These results were consistent with the literature supporting the association between tooth loss and decreased cognitive function [5, 9–19]. According to Stewart and Hirani (2007) the localized inflammatory reaction associated with periodontal disease could lead to a state of chronic low-grade systemic infection and an increase in the cytokines reaching the brain [16]. Moreover, it has been noted that individuals with fewer teeth are at a greater risk of developing nutritional deficiencies, especially B vitamins, which play a major role in the pathogenesis of dementia and cognitive decline [5, 40–42]. According to animal models studies, loss of teeth was associated with neuroanatomical and chemical changes that may eventually have a negative effect on learning and memory [18, 19]. It is important to consider the likelihood of a reversed casualty, whereby individuals with poor cognition may have a lower ability to exact oral hygiene measures, such as tooth brushing and denture care, which ultimately leads to poor oral health [54, 55].

Additionally, a greater prevalence of low SMMSE scores was observed in persons with impaired chewing ability (*P* < 0.05) (Figure 2). With the use of functional magnetic resonance imaging (fMRI) and positron emission tomography (PET), multiple studies have observed an increase in cortical blood flow [20–26] and a rise in oxygen level in the prefrontal cortex and hippocampus [6, 9] during mastication. Chewing has also been noted to stimulate increased cardiac activity, suggesting greater sympathetic stimulation, which increases blood glucose levels and arousal when undertaking a cognitive task [25, 26]. Furthermore, according to Teixeira et al. there was an increased performance in relation to memory retrieval when elderly people aged 60–70 years used chewing gum [26]. In conjunction with the former, animal model studies revealed a causal relationship; occlusal hypofunction caused degenerative changes in periodontal mechanoreceptors, which in effect lead to the suppression of sensory stimulation from the periodontal ligaments during mastication and poor performance in memory and learning tests [56–59].

Although a lower number of remaining teeth and decreased chewing ability were significantly correlated with lower SMMSE scores, it must be noted that individuals from the elderly population usually present with other risk factors which may have affected their cognitive function. Hence, the decreased number of remaining teeth and impaired chewing ability may not alone lead to cognitive decline but may be markers of comorbidities [60, 61].

In this pilot study, no significant relationship was found between denture presence and cognitive function (*P* > 0.05), which may be justified by the insignificant number of denture wearing individuals within the study sample.

A significant difference in SMMSE scores was noted between nationals and expatriates (*P* > 0.05), which may be related to the nationals' difficulty in accessing schools until the late 1950's [62].

Education was significantly correlated with higher SMMSE scores (*P* < 0.05). These results are in accordance with the cognitive reserve hypothesis, which assumes some aspects of life experiences such as education and knowledge act as markers of cognitive reserve and protect against age-associated cognitive decline in later life [6].

Limitations of the present study merit consideration. The primary limitation arises due to the nature of cross-sectional study; hence a casual inference is difficult to make regarding the relationship between teeth remaining, chewing ability, denture presence, and poor cognitive function. Future studies with longitudinal designs would help investigate these associations. Additionally, cognitive function was assessed using only the SMMSE and although it is the most widely used means for examining cognitive function, the education level of an individual may influence it. Finally, due to the small sample size and since most of the participants were volunteers residing in elderly homes, the results of this pilot study may not represent the general population.

## 5. Conclusion

In conclusion, this pilot study revealed a significant finding whereby persons with fewer number of remaining teeth and impaired chewing ability demonstrated poor cognitive ability. The results draw attention to the possible role a person's oral health may play on their cognitive function. However, the interpretation of the results was hindered by the lack of longitudinal observation and the use of a single examination for cognitive function. Therefore, future research investigating the associations is required and the limitations need to be addressed.

## Figures and Tables

**Table 1:** Demographic characteristics of the subjects.

	Control	Low SMMSE score	Pearson's *r*	*P* value
	*n* = 19	*n* = 31		
Age	67.7 ± 5.4	73.5 ± 11.8	−0.277	0.052^*∗*^
Gender				
Male	11 (57.9)	14 (45.2)	0.124	0.382^†^
Female	8 (42.1)	17 (54.8)		
Nationality				
UAE	4 (16.0)	21 (84.0)	−0.453	0.001^†^
Expatriate	15 (60.0)	10 (40.0)		
Education				
Uneducated	3 (10.3)	24 (16.7)	0.600	0.001^†^
Educated	16 (8.7)	1 7 (14.3)		
Health				
Hypertension	7 (30.4)	16 (69.6)	−0.144	0.387^†^
Diabetes	7 (29.2)	17 (70.8)	−0.175	0.255^†^
Smoking				
Nonsmoker	4 (21.1)	15 (16.0)	0.108	0.459^†^
Smoker	4 (5.0)	27 (26.0)		

SMMSE: Standardized mini-mental state examination; ^†^Chi Square, as no., %; ^*∗*^Unpaired  *T*-Test, as mean, standard deviation.

**Table 2:** Summary statistics of oral health variables and SMMSE scores.

	Control	Low SMMSE score	Pearson's *r *	*P* value
	*n* = 19	*n* = 31		
Teeth remaining				
(0–10)	3 (13.6)	19 (86.4)	0.465	0.04^†^
(11–21)	5 (45.5)	6 (54.5)		
(22–32)	11 (64.7)	6 (35.3)		
Chewing ability				
Competent	13 (52.4)	11 (45.8)	0.320	0.04^†^
Impaired	6 (23.1)	20 (76.9)		
Denture				
Present	2 (28.6)	5 (71.4)	0.078	0.695^†^
Absent	17 (39.5)	26 (60.5)		

SMMSE: Standardized mini-mental state examination; ^†^Chi Square, as no., %.
